# A randomized controlled trial comparing lithium plus valproic acid versus lithium plus carbamazepine in young patients with type 1 bipolar disorder: the LICAVAL study

**DOI:** 10.1186/s13063-019-3655-2

**Published:** 2019-10-26

**Authors:** Giovani Missio, Doris Hupfeld Moreno, Frederico Navas Demetrio, Marcio Gerhardt Soeiro-de-Souza, Fernando dos Santos Fernandes, Vivian Boschesi Barros, Ricardo Alberto Moreno

**Affiliations:** 1 Department of Psychiatry, Mood Disorder Unit (GRUDA), University of São Paulo School of Medicine, Rua Dr. Ovídio Pires de Campos, 785, São Paulo, SP 05403-010 Brazil; 2 University of São Paulo School of Public Health, Av. Dr. Arnaldo, 715, São Paulo, SP 01246-904 Brazil

## Abstract

**Background:**

Treatment of bipolar disorder (BD) usually requires drug combinations. Combinations of lithium plus valproic acid (Li/VPA) and lithium plus carbamazepine (Li/CBZ) are used in clinical practice but were not previously compared in a head-to-head trial.

**Objective:**

The objective of this trial was to compare the efficacy and tolerability of Li/VPA versus Li/CBZ in treating type 1 BD in any phase of illness in young individuals.

**Methods:**

LICAVAL was a randomized, unicenter, open-label, parallel-group trial that was conducted from January 2009 to December 2012 in a tertiary hospital in São Paulo, Brazil. Participants were between 18 and 35 years old and were followed up for 2 years. Our primary outcome was the number of participants achieving/maintaining response and remission during the acute and maintenance phases of BD treatment, respectively. Other outcomes assessed were symptom severity and adverse events throughout the study. In the analysis of the primary outcome, we compared groups by using a two-way repeated measures analysis of variance and estimated effect sizes by using Cohen’s d.

**Results:**

Of our 64 participants, 36 were allocated to Li/VPA and 28 to Li/CBZ. Our sample was composed predominantly of females (66.6%) and the average age was 27.8 years. A total of 27 (45.0%) participants had depression, 17 (28.3%) had mania/hypomania, and 16 (26.7%) had a mixed state. We found no between-group differences in CGI-BP (Clinical Global Impression Scale modified for use in bipolar disorder) scores (*P* = 0.326) or in any other outcome. Side effects differed significantly between groups only in the first week of treatment (*P* = 0.021), and there were more side effects in the Li/VPA group. Also, the Li/VPA group gained weight (+2.1 kg) whereas the Li/CBZ group presented slight weight loss (−0.2 kg).

**Conclusion:**

Our study suggests that Li/VPA and Li/CBZ have similar efficacy and tolerability in BD but that Li/CBZ might have metabolic advantages in the long term.

**Trial registration:**

ClinicalTrials.gov identifier: NCT00976794. Registered on September 9, 2009.

## Introduction

Effective treatment of bipolar disorder (BD) should treat and prevent mood episodes [1]. Ideally, these goals are to be achieved with monotherapy. However, more than 80% of patients with BD require drug combinations [2]. A possible strategy is to combine mood stabilizers, such as lithium plus valproic acid (Li/VPA) and lithium plus carbamazepine (Li/CBZ) [3].

These combinations are common in clinical practice and perform better than monotherapy in some patients with BD. However, their comparative efficacy is unknown [4, 5].

Li/VPA is a well-established combination. In the landmark BALANCE trial, Li/VPA had more efficacy than VPA alone in maintenance BD treatment [6]. Other trials have reported advantages of Li/VPA versus monotherapy in specific bipolar populations [7–9]. For instance, in a small study, Li/VPA was associated with marked/moderate improvement in eight of nine patients with refractory rapid cycling BD [7].

Li/CBZ also led to increased response rates in rapid cycling BD compared with Li or CBZ alone, according to Denicoff et al. [10]. This combination also has shown benefit among Li non-responders. In this population, Li/CBZ has been associated with fewer relapses and hospitalizations [11–14]. Also, observational studies associated Li/CBZ with early improvement compared with Li alone [15].

Besides being efficacious, Li/VPA and Li/CBZ are generally safe and well tolerated. [5, 16]. As these drugs are metabolized by different pathways, they lack relevant pharmacokinetic interactions [5, 17]. Also, their combination might be more suitable than antipsychotics in the long term. Although antipsychotics are efficacious in BD, their long-term use is problematic because of metabolic and neurological side effects [18].

Side effects were more frequent with Li/VPA and Li/CBZ than with VPA or CBZ alone in some studies [13]. However, in other studies, these combinations were associated with similar or increased tolerability versus monotherapy [5, 6, 12, 19]. This might have occurred because Li/VPT and Li/CBZ have a synergic action, sometimes allowing lower doses of each drug [20]. Another possible explanation is that side effects of one drug were counteracted by those of the other. For instance, Li might reduce CBZ-induced neutropenia [16].

An additional advantage of Li/VPA and Li/CBZ is that, unlike many first-line BD treatments, these drugs are cheap and widely available. For instance, patients with BD in Brazil’s public health system receive Li, VPA, CBZ, haloperidol, and clorpromazine. Other drugs are usually unavailable for underserved populations, as they can only be obtained out-of-pocket, in academic settings, or under special authorization requests [21, 22].

In summary, Li/VPA and Li/CBZ have a favorable efficacy/safety profile in BD and are of particular importance in underdeveloped countries. However, these combinations were not previously compared in a head-to-head clinical trial. To address this gap in the literature, we designed the LICAVAL study to compare the efficacy and tolerability of Li/VPA versus Li/CBZ in BD.

## Methods

### Study design

LICAVAL was a randomized, unicenter, open-label, parallel-group, equivalence trial comparing the efficacy and tolerability of Li/VPA and Li/CBZ in treating type 1 BD in young individuals. Participants were allocated in a 1:1 ratio. No changes were made to trial design or outcomes after its commencement. Study reporting followed the Consolidated Standards for Reporting of Trials (CONSORT) statement [23].

### Setting

LICAVAL was designed and implemented by the Mood Disorder Unit (GRUDA) at the Department and Institute of Psychiatry, University of São Paulo Medical School (IPq-FMUSP). IPq-FMUSP is a tertiary referral center located in São Paulo (Brazil).

### Ethics

Our study protocol complied with the Declaration of Helsinki [24]. It was approved by the institutional review board at IPq-FMUSP (protocol number: 0820/08) and registered at www.clinicaltrials.gov (ClinicalTrials.gov identifier: NCT00976794). Study participation was voluntary and required written informed consent. No compensation was involved.

### Participants

Research volunteers were self-selected or referred. Recruitment was made through advertisements at the GRUDA website (www.progruda.com) and posters placed at IPq-FMUSP and other psychiatric units. Our recruitment included individuals who were hospitalized or required hospitalization.

Potential participants were pre-screened through phone interviews and further assessed at GRUDA by using the Structured Clinical Interview for the *Diagnostic and Statistical Manual of Mental Disorders-IV-TR* Axis I Disorders (SCID-I) [25].

Eligible participants were between 18 and 35 years old and had a diagnosis of type 1 BD in any phase of illness according to the SCID-I. Patients with clinical and psychiatric comorbidities were included as long as these comorbidities were stable. Exclusion criteria were schizophrenia, schizoaffective disorder, unstable clinical illness, and use of antipsychotics or fluoxetine in the previous month.

### Interventions

Study participants were allocated to receive Li/VPA or Li/CBZ. Participants were assessed weekly in the first month and fortnightly in the second month. Those who had clinical response after 2 months (acute phase of treatment) were assessed monthly for 22 months. Each assessment included all of our diagnostic instruments (see the ‘Outcomes’ section below). These medications should be administered orally. Li was initiated at 300 mg twice daily and increased weekly by 300 mg/day to reach serum levels between 0.4 and 0.8 mEq/L. VPA was initiated at 250 mg twice daily and increased weekly by 500 mg/day to reach serum levels between 50 and 125 μg/mL. CBZ was initiated at 200 mg once a day for the first two days and increased every two days by 200 mg/day to reach serum levels between 8 and 12 μg/mL. Adherence was assessed through pill counts. (Participants were asked to bring their prescribed medication during study visits.)

Other medications allowed at the discretion of psychiatrists were lorazepam 0.5–4 mg/day for insomnia and anxiety and sertraline 25–200 mg/day for bipolar depression. Sertraline was allowed only if depressive symptoms persisted after four weeks of study participation. Participants who finished their participation at the LICAVAL study were followed up at GRUDA for four additional visits over 8 weeks.

### Outcomes

Our primary outcome was the number of participants achieving/maintaining response and remission during the acute and maintenance phases of BD treatment, respectively. Participants’ response in the acute phase of treatment was classified as response (symptom severity reduction of at least 50% with no worsening of symptoms in the opposite pole), no response (symptom severity reduction of less than 50%), and loss of response (response followed by symptom severity reduction of less than 50% in at least one visit or at the physician’s discretion). During the maintenance phase, remission was defined as a symptom severity reduction of at least 75% with no worsening of symptoms in the opposite pole.

According to our study protocol, our secondary out-come was the number of participants achieving BD remission during the maintenance phase of treatment. However, our sample size finishing this phase of the study was insufficient for this analysis. Thus, we decided to include other outcomes of interested of interest as secondary outcomes: (1) BD severity according to the Clinical Global Impressions Scale modified for use in bipolar disorder (CGI-BP) [26]. The CGI-BP is a Likert-type interviewer-rated instrument that measures severity of BD, ranging from 1 (normal, not ill) to 7 (very severely ill). (2) Symptom severity according to the Hamilton Depression Rating Scale (HAM-D) and the Young Mania Rating Scale (YMRS). These are validated and widely used interviewer-rated instruments. The HAM-D is a 17-item scale measuring depression severity [27, 28]. It has Likert-type grading, and scores range from 0 to 52. The YMRS is an 11-item multiple choice scale that measures manic symptom severity in children and young adults [29, 30]. Its scores range from 0 to 60; (3) Time-to-relapse after the acute phase of treatment; (4) use of medications: mean daily doses and serum levels of Li, VPA, and CBZ and mean daily of sertraline; (5) treatment safety and tolerability according to the Udvalg for Kliniske Undersøgelser (UKU) Side Effect Rating Scale, a 48- item clinician-rated structured interview that assesses side effects of psychotropic medications; (6) time-to-discontinuation due to side effects. At baseline, all participants provided sociodemographic information.

### Sample size

Sample size was calculated on the basis of mean within-group differences in CGI-BP scores throughout the study. Using the *t* test, we calculated that a sample size of 50 participants (25 per group) would give us 80% power to detect a standard deviation of 0.8 (at a level of statistical significance of 5%). As our study is an exploratory study, we considered this an appropriate effect size.

### Randomization

We used simple randomization with an allocation ratio of 1:1. Study participants were randomly assigned prior to their first study visit. To prevent selection bias, our random sequence was known by administrative staff only. These staff members were responsible for the following tasks during our study: randomization implementation, visit scheduling, and pill counting. They had no other participation in our study design, implementation, or data analysis.

### Blinding

Our study was open-label. Participants and psychiatrists knew which treatment was being administered. To reduce information bias, outcomes were assessed by blinded raters who had no contact with study records.

### Statistical methods

All analysis were performed by using the IBM SPSS Statistics software version 22 (IBM, Armonk, NY, USA). Significance level was 0.05.

We started our analysis by evaluating central tendency, dispersion, and distribution of all variables. Missing data were imputed through last observation carried forward. Groups were compared with an intention-to-treat approach, but we analyzed only data from participants with at least two visits.

In the evaluation of the primary outcome, we used a two-way repeated measures analysis of variance to analyze between-group differences in scores from the CGI-BP in the acute phase of study. Effect sizes were estimated by using Cohen’s d. Other between-group comparisons were performed by using the *t* test for continuous variables and the Fisher exact test or the chi-squared test for categorical variables.

We also used Kaplan–Meier curves to analyze time-to-relapse and time-to-discontinuation due to side effects in each group. Curves were then compared by using the log-rank test (Mantel–Cox).

## Results

### Participants

**Fig. 1:**
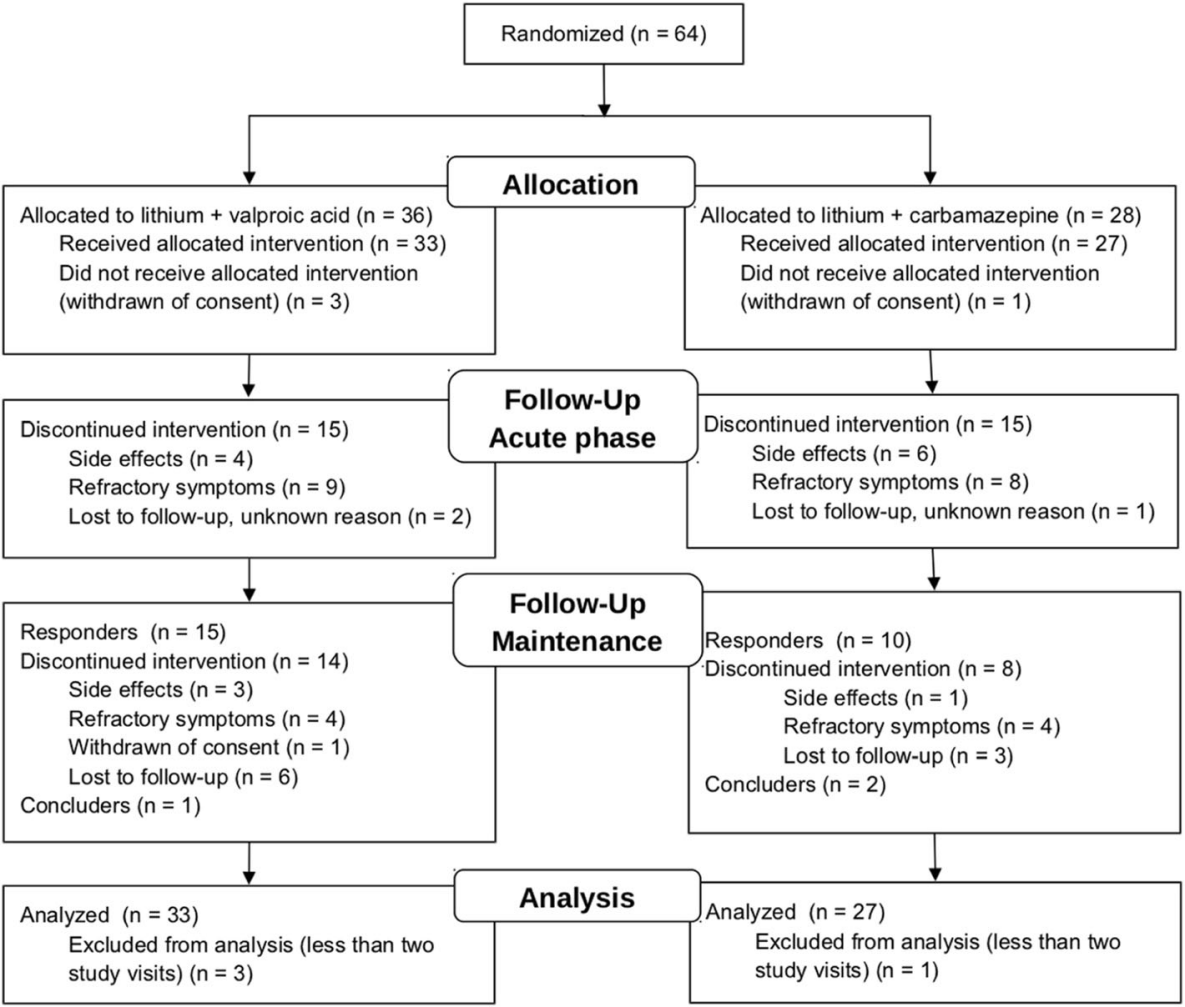
Consolidated Standards for Reporting of Trials (CONSORT) flow diagram

Our study was conducted from January 2009 to December 2012, including patient recruitment. After screening, 64 participants were included: 36 (56.3%) in the Li/VPA group and 28 (43.8%) in the Li/CBZ group (see participant flow in Fig. 1). Owing to withdrawal of consent, four patients were excluded before any treatment (three in the Li/VPA group and one in the Li/CBZ group).

Our sample (Table 1) was composed predominantly of females (66.6%) and the average age was 27.8 years. A total of 27 (45.0%) participants had depression, 17 (28.3%) had mania/hypomania, and 16 (26.7%) had a mixed state.

**Table 1:** Baseline sample characteristics

Variable	Total (*n* = 60)	Li/VPA (*n* = 33)	Li/CBZ (*n* = 27)	*P* value
Female, *n* (%)	40 (66.7%)	26 (78.8%)	14 (51.8%)	0.05 *
Age, mean (standard deviation (SD), range)	27.8 (5.1, 18–35)	27.5 (4.7, 18–35)	28.3 (5.7, 18–33)	0.57 **
Age at illness onset, mean (SD, range)	20.4 (6.9, 5–35)	20.0 (7.1, 5–30)	20.9 (6.7, 13–5)	0.72 **
Weight in kg, mean (SD)	71.3 (14.4)	73.2 (14.1))		0.09 *
Current mood, *n* (%)				
Depression	27 (45.0%)	13 (39.4%)	14 (51.9%)	0.57 **
Mania/hypomania	17 (28.3%)	11 (33.3%)	6 (22.2%)	
Mixed state	16 (26.7%)	9 (27.3%)	7 (25.9%)	
Clinical Global Impressions Scale modified for use in BP, mean (SD, range)				
Overall	3.8 (1.1, 1.0–6.0)	3.8 (1.1, 2.0–6.0)	3.8 (1.0, 1.0–6.0)	0.84 **
Depression	3.3 (1.6, 0.0–6.0)	3.2 (1.5, 0.0–6.0)	3.4 (1.8, 0.0–6.0)	0.62 **
Mania/hypomania	2.8 (1.6, 1.0–6.0)	3.2 (1.7, 1.0–6.0)	2.4 (1.3, 1.0–5.0)	0.07 **
Manic symptoms (Young Mania Rating Scale) scores, mean (SD, range)	20.4 (7.2) (5.0–39.0)	19.3 (7.1, 8.0–35.0)	21.6 (7.3, 5.0–39.0)	0.22 **
Depressive symptoms (Hamilton Depression Rating Scale), mean (SD, range)	11.3 (5.9, 2.0–28.0)	11.5 (6.0, 2.0–24.0)	11.1 (5.9, 4.0–28.0)	0.81 **

Li/VPA: lithium plus valproic acid group

Li/CBZ: lithium plus carbamazepine group

*Determined by the Fisher exact test.

**Determined by the student *t* test.

### Outcomes and estimation

#### Clinical impression

**Fig. 2:**
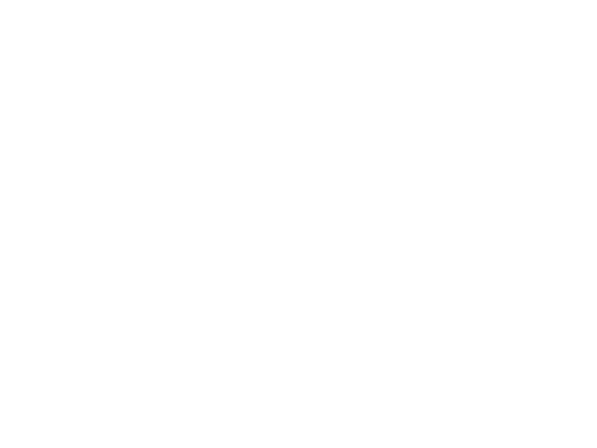
Mean Clinical Global Impression Scale (CGI) scores per group at baseline and after 8 weeks in the LICAVAL study

Study participants had a significant reduction in scores from the CGI-BP, our primary outcome (Fig. 2). Effect sizes were high in the Li/VPA group (*P* = 0.001; d Cohen = 0.80) and average in the Li/CBZ group (*P* = 0.058; d Cohen = 0.53) (Table 2). Between-group differences were not significant (*P* = 0.326).

**Table 2:** Outcome assessment (total and by group)

Variable	Li/VPA group	Li/CBZ group	*P* value
Clinical Global Impressions Scale modified for use in BP (CGI-BP)			0.33**
Week 0, average (standard deviation (SD))	3.8 (1.1)	3.8 (1.1)	
Week 8, average, SD	2.7 (1.6)	3.1 (1.5)	
Within-group difference, *P* value*, Cohen’s d	0.001, 0.80	0.057, 0.53	
CGI-BP - Depression			0.89**
Week 0, average (SD)	3.2 (1.5)	3.5 (1.7)	
Week 8, average (SD)	2.1 (1.1)	2.5 (1.6)	
Within-group difference *P* value*, Cohen’s d	0.005, 0.84	0.014, 0.61	
CGI-BP - Mania			0.18**
Week 0, average (SD)	3.2 (1.7)	2.0 (1.4)	
Week 8, average (SD)	2.4 (1.4)	1.9 (1.0)	
Within-group difference, *P* value*, Cohen’s d	0.001, 0.77	0.152, 0.41	
Depressive symptoms (Hamilton Depression rating Scale)			0.36**
Week 0, average (SD)	19.2 (7.2)	21.6 (7.3)	
Week 8, average (SD)	11.3 (7.2)	11.5 (6.0)	
Within-group difference, *P* value*, Cohen’s d	<0.001, 1.10	<0.001, 1.51	
Manic symptoms (Young Mania Rating Scale)			0.68**
Week 0, average (SD)	11.4 (6.2)	11.1 (5.9)	
Week 8, average (SD)	8.7 (5.3)	7.5 (5.2)	
Within-group difference, *P* value*, Cohen’s d	0.041, 0.47	0.023, 0.65	
Treatment response in 2 months, n (%)	15 (45,5%)	10 (37,0%)	0.61**
Treatment remission in 22 months, n (%)	9 (27.2%)	6 (22.2%)	0.77**

Li/VPA: lithium plus valproic acid group

Li/CBZ: lithium plus carbamazepine group

* Determined by the paired sample *t* test.

** Determined by the two-way repeated measures analysis of variance.

CGI-BP - Depression scores were significantly reduced in both groups. The reduction was higher in the Li/VPA group (*P* = 0.005; d Cohen = 0.84) than in the Li/CBZ group (*P* = 0.014; 0.61), but between-group differences were not significant (*P* = 0.898).

CGI-BP - Mania scores also had a significant reduction in the Li/VPA group (*P* = 0.001; d Cohen = 0.77), and the effect size was large. In the Li/CBZ group, we found a non-significant reduction in CGI-BP scores ((*P* = 0.152; d Cohen = 0.41), and effect sizes were average. Between-group differences were not significant (*P* = 0.175).

#### Symptom severity

**Fig. 3:**
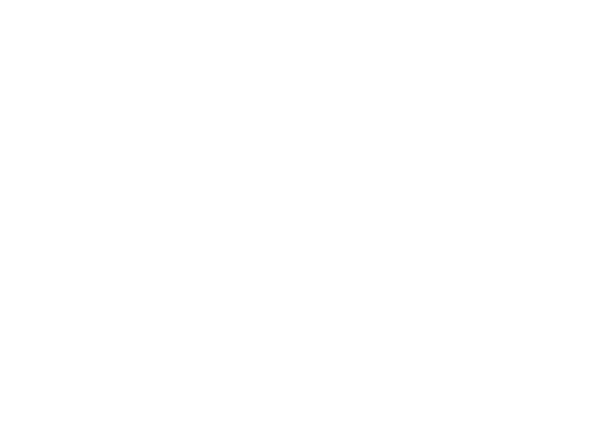
Hamilton Depression Rating Scale and Young Mania Rating Scale - scores per group at baseline and after 8 weeks in the LICAVAL study

The Li/VPA and Li/CBZ groups had a significant reduction in depression scores according to the HAM-D (Fig. 3). Effect sizes were very large. The mean reduction was higher in the Li/CBZ group (*P* <0.001; d Cohen = 1.51) than in the Li/VPA group (*P* <0.001; d Cohen = 1.10), but between-group differences were not statistically significant (*P* = 0.359).

Similarly, both groups had significant reduction in mania scores according to the YMRS. Effect sizes were medium. Differences were higher in the Li/CBZ group (*P* = 0.023; d Cohen = 0.65) than in the Li/VPA group (*P* = 0.041; d Cohen = 0.47), but again between-group differences were not significant.

#### Clinical response and time-to-relapse

Regarding our primary outcome, we found a response rate of 43.3% and no significant between-group differences. In the acute phase of treatment, treatment response was obtained by 15 (45.5%) of 33 participants in the Li/VPA group and 10 (37.0%) of 27 in the Li/CBZ group (*P* = 0.602).

**Fig. 4:**
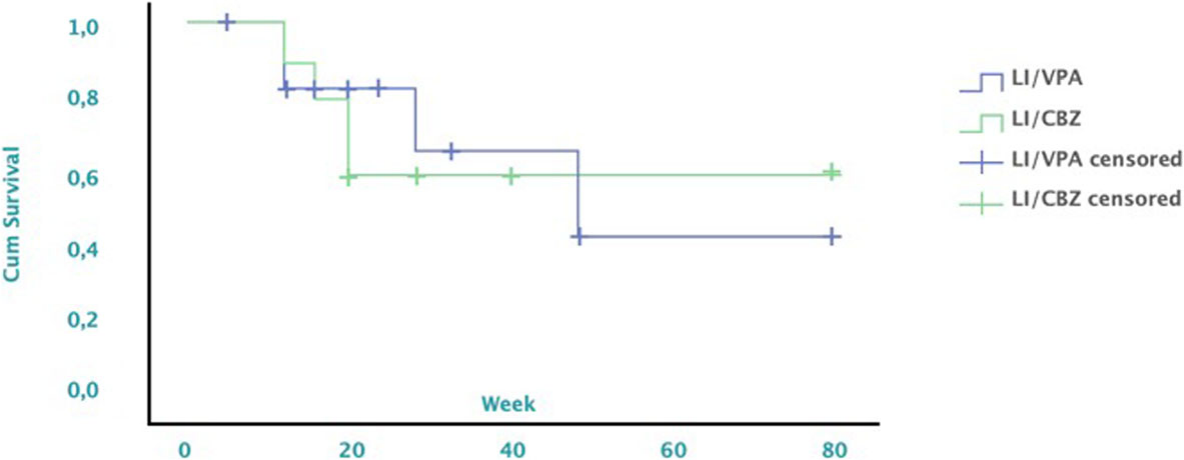
Cumulative survival per group in the LICAVAL study

Responders were followed-up for 22 months, during which 25% had remission of BD symptoms. In this period, remission was achieved by 10 (37.0% of 27) of 15 and 6 (22.2% of 27) of 10 participants in the Li/VPA and Li/CBZ groups, respectively. Time-to-relapse curves were not significantly different between groups (*P* = 0.455) (Fig. 4).

#### Use of medications

Li doses and serum levels were not significantly different between groups (*P* = 0.562). In the Li/VPA group, Li doses were 1024 mg (standard deviation (SD) = 324.3) and serum levels were 0.71 mEq/L (SD = 0.22). In the Li/CBZ group, Li doses were 989.6 mg (SD = 284.8) and serum levels were 0.64 mEq/L (SD = 0.39).

The Li/VPA group had a mean dose of VPA of 1003 mg (SD = 393) and a mean serum level of 70.35 mg (SD = 20.87). The Li/CBZ group had a mean dosage of CBZ of 586 μg/dL (SD = 255) and a mean serum level of 5.66 μg/dL (SD = 1.65).

Ten participants required sertraline, six in the Li/VPA group and four in the Li/CBZ group. Use of sertraline was different between groups only in week 20 (*P* = 0.037). In this week, the Li/CBZ group had a higher mean dosage of sertraline (50.0, SD = 70.7) than the group Li/VPA group. There were no significant differences in use of sertraline in other periods of the study.

#### Safety and tolerability

Side effects differed significantly between groups in the first week of treatment (*P* = 0.021). In this period, the Li/VPA group presented a higher mean number of adverse events per participant than the Li/CBZ group: 3.30 (SD = 4.65) versus 5.63 (4.21), respectively. In other periods of the study, we found no significant between-group differences in adverse events.

According to the UKU Side Effect Rating Scale (Table 3), the Li/VPA group had higher frequencies of fatigue (*P* = 0.015), weight gain (*P* = 0.013), and decreased sexual desire (*P* = 0.036). The Li/CBZ group had increased rates of diarrhea (*P* = 0.001).

**Table 3:** Side effects according to the UKU Side Effect Rating Scale (total and by group)

Side effect	Total, *n* (%)	Li/VPA, *n* (%)	Li/CBZ, *n* (%)	*P* value*
Fatigue	10 (19.5%)	8 (26.7%)	2 (8.3%)	0.02*
Decreased sex drive	8 (11.1%)	7 (20.0%)	1 (4.2%)	0.04*
Diarrhea	9 (17.7%)	2 (6.3%)	7 (29.2%)	0.001*
Weight gain	49 (90.7%)	30 (100.0%)	19 (79.2%)	0.013*

* Determined by the Fisher exact test.

Weight variation from baseline to week 8 was significantly different between groups (*P* = 0.047). In the Li/CBZ group, five participants lost weight. The average weight loss in this group was 0.2 kg (0.44 pounds, *P* value for the within-group difference = 0.001). In the Li/VPT group, participants gained 2.1 kg on average (4.63 pounds, *P* value = 0.03).

Only one serious adverse event occurred during the study: a participant in the Li/CBZ group had severe diarrhea. Given the severity of this adverse event, we decided to withdraw the patient from our study. Patient’s diarrhea remitted after Li/CBZ were discontinued. Despite this, we could not confirm its etiology or relationship with these drugs. We had no reports of Li-associated neurological, thyroidean, or renal adverse events.

A total of 14 participants discontinued treatment because of side effects. Seven were in the Li/VPA group (four in the acute phase and three in the maintenance phase), and seven were in the Li/CBZ group (six in the acute phase and one in the maintenance phase). We found no significant between-group differences in curves of time-to-discontinuation due to side effects (*P* = 0.709).

## Discussion

To our knowledge, this is the first head-to-head comparison of Li/VPA versus Li/CBZ in BD. Our findings indicate that, for young adults with BD, Li/VPA and Li/CBZ have similar efficacy in acute treatment and relapse prevention over 2 years. One strength of our study is that we had participants in all phases of BD, as in a real-world setting.

We found response and remission rates of 43.3% and 25%, respectively, and no significant between-group differences. These rates are lower than those reported in some studies. For instance, in the BALANCE trial, remission occurred in around 45% of participants receiving Li/VPT in a 2-year follow-up [6]. Also, Denicoff et al. reported marked/moderate CGI-BP improvement in around 55% of participants in one year [10]. Our results probably reflect the characteristics of our sample. Participants of LICAVAL were essentially a convenience sample from a tertiary referral hospital. This population tends to be more difficult to treat, explaining our relatively low response and remission rates.

As our study indicates that Li/VPA and Li/CBZ have similar efficacy, choosing between these combinations depends greatly on their tolerability. In acute treatment, Li/CBZ seemed better tolerated by our sample (fewer side effects). Also, surprisingly, the Li/CBZ group had a slight weight reduction over time whereas the Li/VPA group gained weight. Weight gain is frequent in BD treatment and is a cause of treatment abandonment [31, 32]. We are unaware of previous studies associating CBZ with weight loss. In the literature, both CBZ and VPT were associated with slight weight gain whereas Li seems neutral [33].

Based on our findings, we hypothesize that Li/CBZ has a metabolic advantage compared with Li/VPA and perhaps CBZ alone. In the long term, this metabolic advantage might be associated with an increase in treatment adherence and with a decrease in metabolic syndrome and cardiovascular disease rates. Thus, although Li/CBZ and Li/VPA have a similar efficacy in our study, Li/CBZ might be more effective and efficient in treating BD [34]. However, we must acknowledge that our side effect analysis was not corrected for multiple comparisons. Also, LICAVAL was not powered to compare side effect rates between groups.

Despite its relevance, our study has limitations. First, our sample was composed mainly of patients from a tertiary referral center. These patients likely have more severe and refractory BD than the general population, impairing our study external validity. Despite this limitation, our results should be valid for similar populations. Second, we used sequential allocation, a method that can lead to selection bias. Yet we protected our allocation sequence, which was known only by administrative personnel who had no direct participation in our study. Third, our study was open-label, which might have influenced our results. However, our raters were blinded, reducing bias in outcome assessment. Fourth, we allowed the use of sertraline in depressed participants, which might have impacted the effect of Li/VPA or Li/CBZ in our participants. We believe this did not affect our results, as sertraline use was not different between study groups. Fifth, only three participants completed the maintenance phase of our study. As previously stated, this prevented us from analyzing treatment outcomes during this phase of BD treatment. Finally, although we calculated a sample size to achieve 80% power, larger samples might have shown between-group differences.

In conclusion, the results of the LICAVAL study suggest that Li/VPA and Li/CBZ have similar efficacy and tolerability in BD but that Li/CBZ might have metabolic advantages in the long term. Our results should aid evidence-based decision-making regarding BD treatments, especially in underdeveloped countries.

## Data Availability

Not applicable.

## Abbreviations

BD: Bipolar disorder
CBZ: Carbamazepine
CGI-BP: Clinical Global Impression Scale modified for use in bipolar disorder
GRUDA: Mood Disorder Unit
HAM-D: Hamilton Depression Rating Scale
IPq-FMUSP: Psychiatry Institute of University of São Paulo School of Medicine
Li: Lithium
Li/CBZ: Lithium plus carbamazepine
Li/VPA: Lithium plus valproic acid
SCID-I: Structured Clinical Interview for the *Diagnostic and Statistical Manual of Mental Disorders-IV-TR* Axis I Disorders
SD: Standard deviation
UKU: Udvalg for Kliniske Undersøgelser
VPA: Valproic acid
YMRS: Young Mania Rating Scale
